# Multidrug resistance and carbapenemase producing *Enterobacteriaceae* isolated from clinical samples, Addis Ababa, Ethiopia: A cross-sectional study

**DOI:** 10.1371/journal.pone.0356234

**Published:** 2026-08-13

**Authors:** Yordanos Getachew, Tamirat Tamiru, Mekdelawit Fisseha, Sami Sebri, Tadesse Shume, Regasa Diriba, Kassu Desta

**Affiliations:** 1 Department of Microbiology Laboratory, Wudassie Advanced Medical Laboratory, Addis Ababa, Ethiopia; 2 Department of Vaccine, Diagnostic, Medical device Research and Development, Armauer Hansen Research Institute, Addis Ababa, Ethiopia; 3 Department of Medical Laboratory Sciences, College of Health Sciences, Addis Ababa University, Addis Ababa, Ethiopia; Debre Markos University, ETHIOPIA

## Abstract

**Background:**

Multidrug-resistant *Enterobacteriaceae* (MDRE) is among the leading causes of hospital-acquired infections, resulting in prolonged hospitalization, increased healthcare costs, and mortality. Carbapenem are often used as last-resort antibiotics; however, the emergence of carbapenemase-producing *Enterobacteriaceae* (CPE) has severely limited treatment options. In Ethiopia, data on their burden from routine diagnostic laboratory settings remain limited. This study assessed the prevalence of multidrug-resistant and carbapenemase-producing *Enterobacteriaceae* among clinical isolates in Addis Ababa.

**Methods:**

A laboratory-based cross-sectional study was conducted from January 2, 2025, to April 2, 2025 on clinical specimens referred to Wudassie Advanced Medical Laboratory in Addis Ababa, Ethiopia. A total of 1105 clinical specimens collected through convenience sampling method were processed. Bacterial identification was performed using matrix-assisted laser desorption/ionization time-of-flight mass spectrometry (MALDI-TOF MS) (EXS 3000, Zybio Inc., China). Antimicrobial susceptibility testing was conducted using the Kirby–Bauer disk diffusion method according to Clinical and Laboratory Standards Institute (CLSI 2024) guidelines. Carbapenemase production was confirmed using the modified Carbapenem Inactivation Method (mCIM). Data were analyzed using SPSS version 26.

**Results:**

Of the 1105 clinical specimens, 288/1105 (26.1%) showed bacterial growth. *Enterobacteriaceae* accounted for 156/288 (54.2%) of the isolates. The most common isolates were *Escherichia coli* 89/156 (57.1%) and *Klebsiella pneumoniae* 47/156 (30.1%). Overall, 123/156 (78.8%) of *Enterobacteriaceae* were multidrug resistant. Carbapenem resistance was detected in 35/154 (22.7%) of isolates, and 30/154 (19.5%) were confirmed as carbapenemase producers. *Klebsiella pneumoniae* 24/30 (80%) was the predominant carbapenemase-producing organism. Most CPE isolates were recovered from urine specimens, followed by wound and sputum samples.

**Conclusion:**

This study demonstrates a high prevalence of multidrug-resistant and carbapenemase-producing *Enterobacteriaceae* in Addis Ababa. The findings highlight the urgent need for strengthened antimicrobial stewardship programs, early detection strategies, and enhanced infection prevention and control measures in the private health facilities.

## Introduction

Antimicrobial resistance (AMR) has become one of the most serious global public health threats, compromising the effectiveness of modern medicine. The inappropriate and excessive use of antimicrobial agents has accelerated the emergence and spread of resistant pathogens, leading to increased mortality, prolonged hospitalization, and substantial healthcare costs [1].

Among resistant organisms, multidrug-resistant (MDR) *Enterobacteriaceae* represent a major public health concern due to their widespread occurrence in both hospital and community settings. These pathogens commonly exhibit resistance to multiple antibiotic classes, severely limiting therapeutic options [2]. Carbapenem are considered last-resort antibiotics for severe infections caused by extended-spectrum β-lactamase (ESBL)-producing Gram-negative bacteria. However, the global emergence and spread of carbapenemase-producing *Enterobacteriaceae* (CPE) have significantly reduced the effectiveness of these agents [3].

The global burden of AMR remains substantial. In 2019, bacterial AMR was associated with an estimated 4.95 million deaths worldwide, with the highest burden reported in sub-Saharan Africa [4]. Carbapenem-resistant *Enterobacteriaceae*, particularly Carbapenem-resistant *Klebsiella pneumoniae*, are of major concern, with reported mortality rates ranging from 37.2% to 42.1% and a high number of infections and deaths globally [5].Despite this burden, treatment options remain limited, and optimal therapeutic strategies for Carbapenem-resistant infections are still evolving [6].

The overall pooled prevalence of multidrug resistance in Ethiopia was 70.5% [7], indicating a substantial burden of antimicrobial resistance among bacterial isolates. Furthermore, the pooled prevalence of carbapenemase-producing Enterobacteriaceae was 5.44% [8], highlighting the emergence of highly resistant pathogens that pose a significant challenge to effective antimicrobial therapy and public health.

However, most available evidence is derived from public healthcare institutions; data from routine diagnostic laboratory settings remain limited. Generating updated evidence from such settings is essential to support antimicrobial stewardship and infection prevention strategies.

Therefore, this study aimed to determine the prevalence of multidrug-resistant and carbapenemase-producing *Enterobacteriaceae* isolated from clinical specimens in Addis Ababa, Ethiopia.

## Materials and methods

### Study settings

A laboratory-based cross-sectional study was conducted from January 2, 2025, to April 2, 2025 on specimens referred to Wudassie Advanced Medical laboratory (WAML), a private accredited diagnostic laboratory with five branches found in Addis Ababa, Ethiopia. Clinical samples were collected at all branches and processed at Piassa branch. 9.028373° N, 38.751977° E.

### Population

#### Source population.

All patients whose specimen referred to Wudassie Advanced Medical Laboratory during the study period were source populations.

### Study population

All patients who were suspected of bacterial infections and whose specimen were referred to WAML for culture were the study population

### Inclusion criteria

- Specimens from all age group patients who were suspected of having different bacterial infections.

### Exclusion criteria

- Patients who took antibiotics within 7 days of specimen collection.

### Study variables

### Dependent variable

- Profile of Enterobacteriaceae
- Prevalence of carbapenemase producers

### Independent variable

- Age
- Sex
- Specimen type
- Health facility types
- Patient admission status

### Sample size determination and Sampling technique

The minimum sample size was calculated using a single population proportion formula based on a previously reported MDR prevalence of 68.6% [9],95% confidence interval, 5% margin of error.


$$\begin{array}{c} n=\frac{z_{\frac{\alpha }{\text{2}}}pq}{d^{\text{2}}} \end{array}$$

Based on this the calculated sample size was 331, however a total of 1105 eligible specimens obtained during the study period were included to increase representativeness and maximize bacterial recovery. Convenience sampling method was employed.

### Data collection

Socio demographic data and data regarding admission status and facility type were collected by trained laboratory personnel through pre-structured data collection tool.

### Sample collection and analysis

Specimens, including urine, blood, sputum, wound swabs, stool, and other body fluids, were collected by health professionals working at referring health facilities using standard protocols and transported promptly to the laboratory [10]. Blood sample was collected using prepared sterile blood culture bottles. Two bottles were collected for adults with 8–10 ml blood in each to increase the sensitivity of detection and to rule of contaminant growth from single bottle. In pediatric, 1–2 ml of blood in pair of bottles was collected and processed. All specimens were inoculated on Blood broth, MacConkey agar, Blood agar, XLD agar plates according to their specific growth requirement and incubated at 37°C for 18–24 hours. All isolates were characterized by colony appearance, lactose fermentation and identified by matrix-assisted laser desorption ionization-time of flight mass spectrometry (MALDI TOF)(EXS 3000, Zybio Inc., China) [11].

### Identification with MALDI-TOF

The sample is prepared by mixing or coating it with a solution containing an energy-absorbing organic compound known as a matrix. As the matrix dries and crystallizes, the sample becomes co-crystallized within it. During analysis, a laser beam automatically ionizes the sample embedded in the matrix and produces singly protonated ions from the analytes present in the sample. The protonated ions are accelerated under a constant electric potential, causing them to separate based on their mass-to-charge ratio (m/z). These charged analytes are then detected and measured using a time-of-flight (TOF) analyzer. In MALDI-TOF analysis, the m/z ratio is determined by measuring the time it takes for each ion to travel through the flight tube. This time-based data is used to generate a unique spectrum known as a peptide mass fingerprint (PMF), which is then compared to a reference library to identify the bacterial species present in the sample [11].

### Antibiotic susceptibility testing

The antimicrobial susceptibility testing isolates were performed by using the Kirby–Bauer disk diffusion technique as described by the Clinical and Laboratory Standards Institute (CLSI 24) [12]. From the growth in the agar medium, three to four selected colonies were transferred to a tube containing 5 ml of normal saline (0.85%) and mixed gently to make a homogenous suspension. The turbidity of the suspension was adjusted to a McFarland 0.5 standard. A sterile cotton swab was used to streak the plates. The excess suspension was removed by gentle pressing and rotation of the swab against the tubes inside the wall surface. The swab was used to distribute the bacteria evenly over the entire surface of the Mueller–Hinton agar (MHA). The inoculated plates were kept at room temperature to dry for 3–5 min. With sterile forceps, the following concentration of antibiotic discs was put onto the surface of MHA; the plates were incubated at 37°C for 24 h. The antibiotic discs used in this study were ampicillin: (AMP:10 μg), amoxicillin-clavulanic acid (AMC: 20/10 μg), ceftriaxone (CRO:30 μg), cefotaxime (CTX: 30 μg), ceftazidime (CAZ: 30 μg), cefepime (FEP: 30 μg), meropenem (MER: 10 μg), gentamicin (GEN:10 μg), amikacin (30 μg) ciprofloxacin (CIP: 5 μg:), nitrofurantoin (NI:300 μg) sulfamethoxazole-trimethoprim (SXT: 3.75/1.25 μg), and chloramphenicol (C) (30 µg), (using commercially available Oxoid, Basingstoke, England).

### Modified Carbapenem inactivation method

Carbapenem resistant isolates were confirmed for carbapenemase enzyme production by modified Carbapenem inactivation method (mCIM). A fresh bacterial colony was taken using a 1 µL loop and suspended in 2 mL of tryptic soy broth, and then meropenem disk was placed into the suspension, and incubated at 35°C for 4 hours ± 15 minutes. Carbapenem sensitive *E. coli* ATCC 25922 was used to make suspension of 0.5 McFarland and inoculated on MHA plate as standard antimicrobial sensitivity testing, and within 15 minutes of inoculation, meropenem disk that has been incubated with the test organism was taken out and placed on to the inoculated MHA plate, and incubated over night at 37^0^c.The next day, a zone of inhibition between 6–15 mm was interpreted as carbapenemase producer, zone of inhibition >19mm as non carbapenemase producers. A zone of inhibition between 16 and 18 mm around a disk with distinct colonies inside zone of inhibition was considered as positive test thus carbapenemase producer but a clear zone of inhibition between 16 and 18 mm around a disk was considered indeterminate thus the purity of the *E. coli* ATCC 25922 and integrity of the meropenem disks was verified and the test was repeated [12].

### Data quality assurance

To ensure data quality, the data collection form was pre-tested and the data that has been collected was thoroughly checked on spot and daily for their completeness, accuracy, and clarity. All of the pre-analytical, analytical and post analytical steps were carried out in strict adherence to the Standard Operating Procedures (SOPs) of the microbiology laboratory at Wudassie Advanced Medical Laboratory Sterility of the culture media was checked with indicator tape and by incubating 3–5% of the batch at 37 °C for 24 hours. Media performance was further evaluated by inoculating with control strains. The reference bacterial strains *K. pneumoniae* ATCC BAA-1705 as positive and *K. pneumoniae* ATCC BAA-1706 as negative control isolates were used as a control in each batch of mCIM. Moreover for long-term preservation, purified bacterial cultures were maintained tryptic soy broth supplemented with 20% glycerol at −80°C and were sub-cultured monthly to maintain viability.

### Data analysis and interpretations

Data was entered using Epi-data version 4.6 and analyzed using Statistical Package for the Social Sciences (SPSS) software, version 26. Descriptive statistics were used to summarize the data and results were presented as frequencies and percentages.

### Operational Definitions

**Multi Drug Resistance:** MDR is defined as non-susceptibility to at least one agent in three or more antimicrobial categories [13].

**Carbapenemase production:** is defined as bacteria that have resisted Carbapenem groups of drugs and the isolate is Positive for carbapenemase production by a phenotypic method [14].

### Ethics Approval and Consent to Participate

Ethical approval for this study was obtained from the Departmental Research and Ethics Review Committee (DRERC), Department of Medical Laboratory Sciences, College of Health Sciences, Addis Ababa University, Addis Ababa, Ethiopia (Protocol number: **DRERC/782/24/MLS/**). The study was conducted in a stand-alone diagnostic laboratory using routine clinical specimens submitted for diagnostic purposes. The investigators had no direct contact with patients, and no personal identifiers were collected. Because individual patient consent was not feasible and the study involved minimal risk, the requirement for informed consent was waived by the ethics committee. Institutional permission was obtained prior to commencement of the study. To ensure confidentiality, participant data was secured by password and unauthorized persons had no access to the data. Findings of the study were communicated to physicians.

## Results

### Socio-demographic characteristics of the study participants

A total of 1105 patients who met the eligibility criterion were included. Among these more than half (612/1105, 55.4%) were male. The median and standard deviation of age were 44 and 25.5 respectively. About 313/1105 (28.3%) of the patients were above 60 years and 841/1105 (76.1%) of the patients were referred from private health facility, while 872/1105 (78.9%) of them were admitted patients. Blood specimen holds majority of the specimen 439/1105(39.7%) followed by urine specimen 353/1105 (31.9%). (Table 1).

**Table 1:** Socio-demographic and other characteristics of patients, Addis Ababa, Ethiopia, 2025.

Variables	Category	Frequency	Percent
Age (years)	<1	84	7.6
	1-15	122	11.0
	16-30	167	15.1
	31-45	208	18.8
	46-60	211	19.1
	>60	313	28.4
Sex	Female	493	44.6
	Male	612	55.4
Facility type	Private	841	76.1
	Public	264	23.9
Ward	Inpatient	872	78.9
	Outpatient	233	21.1
Specimen type	Blood	439	39.7
	Urine	353	31.9
	Wound	110	10.0
	CSF	45	4.1
	Body fluid	89	8.0
	Sputum	34	3.1
	Stool	35	3.2

### Bacterial isolates from clinical samples

From 1105 different clinical specimens that were inoculated, 288/1105 (26.1%) of them had growth and of this 195/288 (67.7%) were Gram negative bacteria from which 156/288 (54.2%) of them were *Enterobacteriaceae*, 39/288 (13.5%) of them were non fermentative Gram-negative rods. The remaining 90/288(31.3%) of them were gram positive bacteria and the rest, 3/288 (1.0%) isolates were fungal. From *Enterobacteriaceae* isolates, half (79/156, 50.6%) of them were isolated from females and nearly one-third (55/156, 35.3%) of isolates were from patients above 60 years. Regarding isolates, *E. coli* (9/156, 57.1%) accounts the most abundant bacterial isolates followed by *K. pneumonia* (47/156, 30.1%). Majority, (95/156, 60.9%) of the *Enterobacteriaceae* were isolated from urine followed by wound (25/156, 16%) and sputum (13/156, 8.3%). (Table 2).

**Table 2:** Distribution of isolates from different specimen, Addis Ababa, Ethiopia.

Isolate(number)	Blood N (%)	Urine N (%)	Wound N (%)	CSF N (%)	Body fluid N (%)	Sputum N (%)	Stool N (%)	Total N (%)
***C. freundii* (n = 3)**	–	2(66.7)	1(33.3)	–	–	–	–	3(1.92)
***E. coli (*n = 89)**	3(3.37)	70(78.7)	9(10.1)	–	6(6.74)	1(1.12)	–	89(57.1)
***E. cloacae* (n = 3)**	–	–	3(100)	–	–	–	–	3(1.92)
***K. oxytoca*(n = 3)**	–	1(33.3)	1(33.3)	–	–	1(33.3)	–	3(1.92)
***K. pneumoniae*(n = 47)**	7(14.9)	18(38.3)	8 (17)	1(2.1)	3(6.4)	10(21.3)	–	47(30.1)
***M. morganii*(n = 2)**	–	1(50)	1(50)	–	–	–	–	2(1.28)
***P. mirabilis* (n = 5)**	–	3(60)	1(20)	–	–	1(20)	–	5(3.2)
***P. vulgaris* (n = 1)**	–	–	1(100)	–	–	–	–	1(0.64)
***Salmonella spp*(n = 2)**	–	–	–	–	1(50)	–	1(50)	2(1.28)
***S.* marcescens (n = 1)**	1(100)	–	–	–	–	–	–	1(0.64)
**Total (n = 156)**	**11(7.05)**	**95(60.9)**	**25(16)**	**1(0.64)**	**10(6.4)**	**13(8.3)**	**1(0.64)**	**156(100)**

Body fluid: Pleural fluid, Peritoneal fluid, Synovial fluid, Amniotic fluid

### Antimicrobial resistance patterns of the bacteria isolates

Among the 89 E. coli isolates, the majority (85/89, 95.5%), were resistance to ampicillin, while almost two-third (62/89, 69.7%) of them showed resistance to ciprofloxacin; However, most of E. coli (85/89, 95.5%) isolates were susceptible to meropenem. On the other hand, from 47 K. pneumoniae isolates, majority (44/47, 93.6%) were resistant to co-trimoxazole, followed by, 37/47 (78.7%) ciprofloxacin and gentamicin, 28/47 (59.6%) of K. pneumoniae isolates were resistant to meropenem. Overall, ampicillin demonstrated the highest resistance rate 150/156 (96.1%), followed by amoxicillin–clavulanic acid 147/156 (95.5%). In contrast, meropenem had the lowest resistance rate 35/154 (22.7%), followed by nitrofurantoin 30/95 (31.6%). (Table 3**).**

**Table 3:** Distribution of antimicrobial resistance patterns of bacteria isolates to different antibiotics, Addis Ababa, Ethiopia.

Isolate number	AMP N (%)	AMC N (%)	CRO N (%)	CTZ N (%)	FEP N (%)	SXT N (%)	CIP N (%)	AK N (%)	CN N (%)	NI N (%)	C N (%)	MEM N (%)
***C. freundii*** **n = 3**	3 (100)	3 (100)	2 (66.7)	2 (66.7)	2 (66.7)	3 (100)	3 (100)	1 (33.3)	1 (33.3)	–	1 (33.3)	1 (33.3)
***E. coli*** **n = 89**	85 (95.5)	84 (94.4)	60 (67.4)	55 (61.8)	41 (46.1)	71 (79.8)	62 (69.7)	14 (15.7)	30 (33.7)	11 (15.7)	13 (68.4)	4 (4.5)
***E. cloacae*** **n = 3**	3 (100)	3 (100)	2 (66.7)	2 (66.7)	1 (33.3)	2 (66.7)	1 (33.3)	1 (33.3)	2 (66.7)	–	2 (66.7)	–
***K. oxytoca*** **n = 3**	3 (100)	3 (100)	1 (33.3)	1 (33.3)	1 (33.3)	2 (66.7)	2 (66.7)	–	1 (33.3)	1 (33.3)	1 (33.3)	–
***K. pneumoniae*** **n = 47**	47 (100)	46 (97.9)	41 (87.2)	41 (87.2)	39 (83)	44 (93.6)	37 (78.7)	30 (63.8)	37 (78.7)	13 (72.2)	20 (69)	28 (59.6)
***M. morganii*** **n = 2**	2 (100)	2 (100)	2 (100)	2 (100)	2 (100)	2 (100)	2 (100)	1 (50)	2 (100)	1 (50)	1 (50)	–
***P. mirabilis*** **n = 5**	4 (80)	4 (80)	3 (60)	2 (40)	2 (40)	5 (100)	3 (60)	2 (40)	3 (60)	3 (100)	1 (50)	1 (20)
***P. vulgaris*** **n = 1**	1 (100)	1 (100)	1 (100)	1 (100)	1 (100)	1 (100)	1 (100)	1 (100)	1 (100)	–	1 (100)	1 (100)
***Salmonella spp* n = 1(stool)**	1 (100)	NA	NA	NA	NA	–	–	NA	NA	NA	NA	NA
***Salmonella spp* n = 1(body fluid)**	1 (100)	NA	–	NA	NA	–	–	NA	NA	NA	NA	NA
***S.* marcescens** **n = 1**	1 (100)	1 (100)	1 (100)	1 (100)	1 (100)	1 (100)	1 (100)	–	1 (100)	NA	1 (100)	–
**Total n = 156**	**150** **(96.1)**	**147** **(95.5)**	**113** **(72.9)**	**107** **(69.5)**	**90** **(58.4)**	**131** **(84)**	**112** **(71.8)**	**50** **(32.5)**	**78** **(50.6)**	**30** **(31.6)**	**41** **(73.2)**	**35** **(22.7)**

Abbreviations: AMP: ampicillin; AMC: amoxacillin/clavulanate;; CN: gentamicin; SXT: trimethoprim-sulfamethoxazole; CIP: ciprofloxacin; CRO: ceftriaxone; NI: nitrofurantoin; CTZ: ceftazidime; FEP: cefepime; AK: amikacin; C: chloramphenicol; MEM: meropenem; NA: not applicable

### Multi drug resistant *Enterobacteriaceae*

Among the *Enterobacteriaceae* isolates, 123/156 (78.8%) were identified as multidrug-resistant. All isolate of *C.freundii*(3/3), *S.marcescens*(1/1), *M.morganii*(2/2*) and P.vulgaris*(1/1) exhibited multidrug resistance. Multidrug resistance was observed in 67/89 (75%) of *E. coli* and 42/47 (89%) of *K. pneumoniae* isolates. *E. coli* accounted for the largest proportion of MDR isolates (67/123, 54.5%), followed by *K. pneumoniae* (42/123, 34%). As shown in Table 4, only 4/156 (2.5%) of the isolates were susceptible to all tested antibiotic categories, whereas 25/156 (16%) were resistant to seven antibiotic categories, of which 20/25 (80%) were *K. pneumoniae* isolates. (Table 4)

**Table 4:** Distribution of drug resistance patterns to bacterial isolates from different clinical samples, Addis Ababa, Ethiopia.

Isolate (number)	R0 N (%)	R1 N (%)	R2 N (%)	R3 N (%)	R4 N (%)	R5 N (%)	R6 N (%)	R7 N (%)	R ≥ 3 N (%)
***C. freundii* (3)**	–	–	–	1(33.3)	1(33.3)	–	–	1(33.3)	3(100)
***E. coli* (89)**	3(3.4)	9((10.1)	10(11.2)	13(14.6)	30(33.7)	18(20.2)	5((5.6)	1(1.1)	67(75.3)
***E. cloacae* (3)**	–	1(33.3)	–	–	–	1(33.3)	1(33.3)	–	2(66.7)
***K. oxytoca* (3)**	–	1(33.3)	–	–	1(33.3)	1(33.3)	–	–	2(66.7)
***K.pneumoniae*(47)**	–	2(4.3)	3(6.4)	2(4.3)	5(10.6)	4(8.5)	11(23.4)	20(42.5)	42(89.4)
***M. morganii* (2)**	–	–	–	–	–	1(50)	1(50)	–	2(100)
***P. mirabilis* (5)**	–	1(20)	1(20)	–	1(20)	–	–	2(40)	3(60)
***P. vulgaris* (1)**	–	–	–	–	–	–	–	1(100)	1(100)
***Salmonella Spp.* (2)**	1(50)	1(50)	–	–	–	–	–	–	–
***S. marcescens* (1)**	–	–	–	–	–	1(100)	–	–	1(100)
**Total (156)**	**4(2.56)**	**15(9.61)**	**14(8.97)**	**16(10.26)**	**38(24.4)**	**26(16.7)**	**18(11.4)**	**25(16)**	**123(78.8)**

**Notes:** R0-Absence of resistance to any antibiotic category, R1- resistance to one antibiotic category, R2-resistance to two antibiotic categories, R3- resistance to three antibiotic categories, R4- resistance to four antibiotics category, R5- resistance to five antibiotic categories, R6 -resistance to six antibiotic category and R7- is resistance to seven antibiotic categories used for treatment

**Fig 1:**
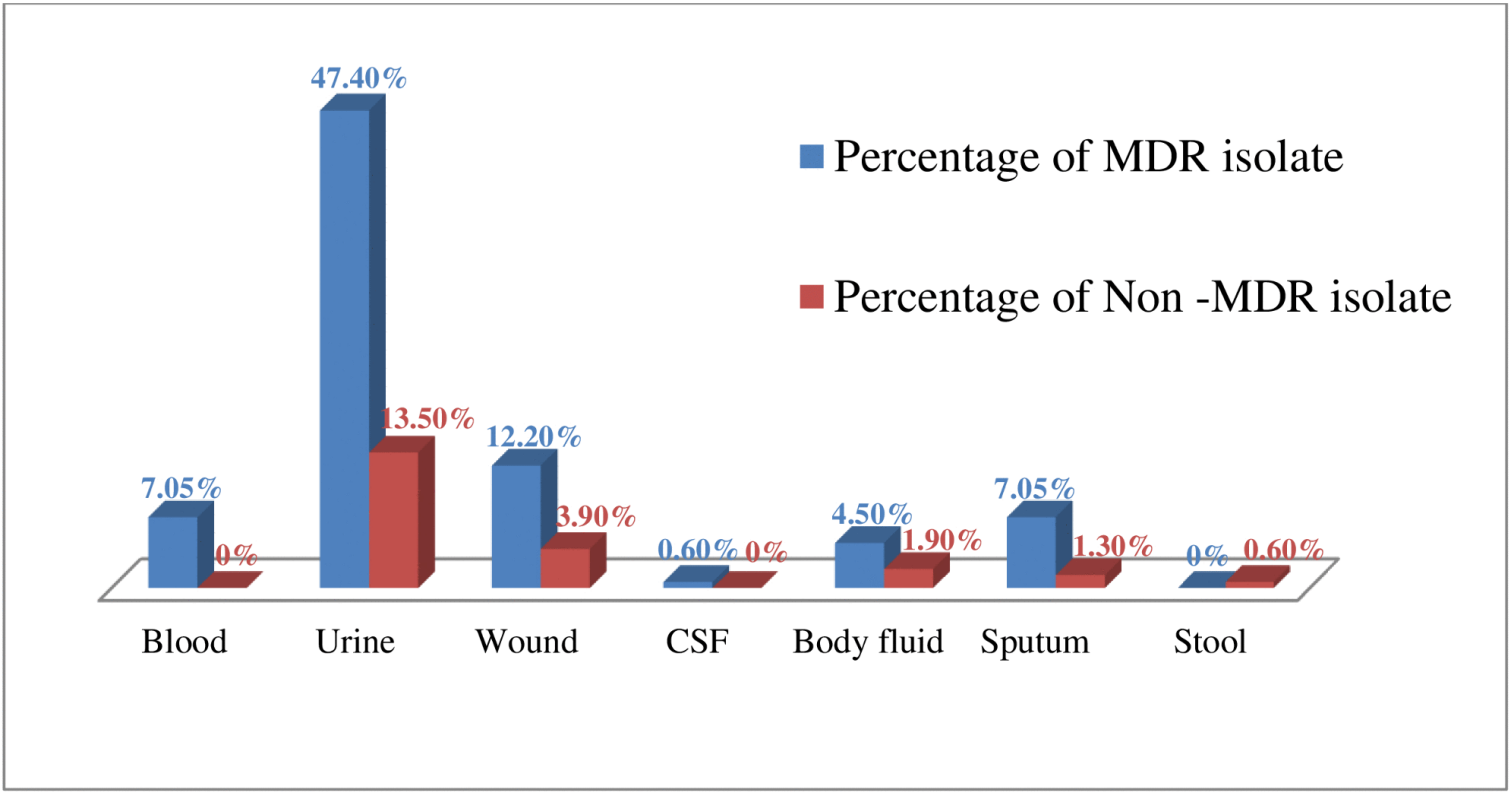
Fraction of multidrug resistant isolate among each clinical specimen. Body fluid includes Pleural fluid, Peritoneal fluid, Synovial fluid, Amniotic fluid.

Larger fractions (74/156, 47.4%) of MDR bacteria were isolated from urine specimen, followed by wound (19/156, 12.2%), blood (11/156, 7.1%), sputum (11/156, 7.1%). Moreover, majority of (104/123, 84.5%) of the MDRE were from admitted patients. (Fig 1).

### Carbapenemase producing *Enterobacteriaceae*

**Fig 2:**
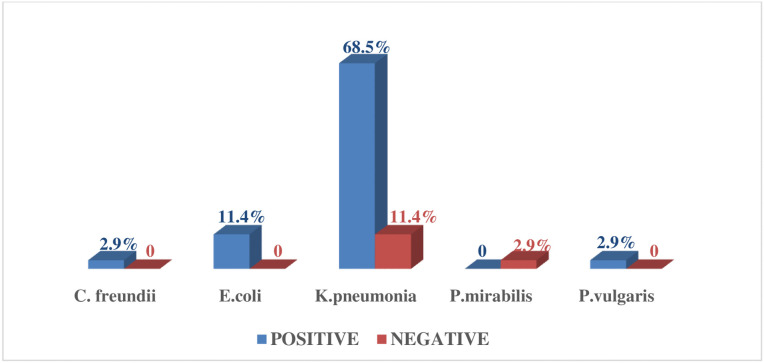
Percentage of carbapenemase production among different bacterial isolates.

From *Enterobacteriaceae* isolated*,* 35/154 (22.7%) of them were Carbapenem resistant*.* Those isolates that has resisted Carbapenem antibiotic were further checked for carbapenemase production by mCIM and 30/35 (85.7%) of them were confirmed to be carbapenemase producers. *K.pneumoniae,* 24/30 (80%) accounts majority of the isolates that produces carbapenemase enzyme (Fig 2).

**Fig 3:**
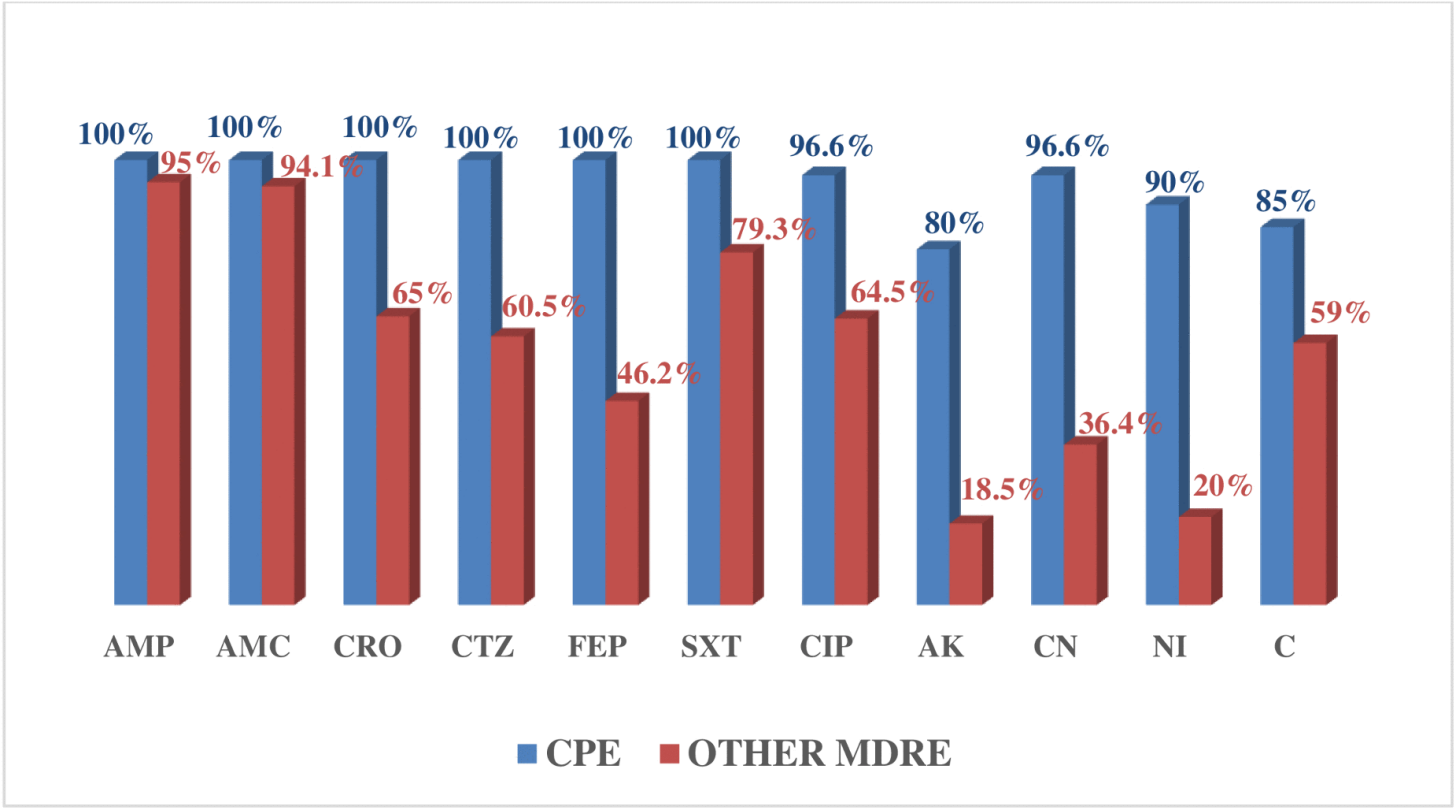
Antimicrobial resistance profile of CPE isolates compared with MDR isolates other than carbapenemase production. AMP: ampicillin; AMC: amoxicillin/clavulanate; CN: gentamicin; SXT: trimethoprim-sulfamethoxazole; CIP: ciprofloxacin; CRO: ceftriaxone; NI: nitrofurantoin; CTZ: ceftazidime; FEP: cefepime; AK: amikacin; C: chloramphenicol; MEM: meropenem.

Drug resistance pattern of CPE isolates were much higher than other MDR isolates with ampicillin (100% versus 94.1%), ceftriaxone (100% versus 65%), ciprofloxacin (96.6% versus 64.5%) and amikacin (80% versus, 18.5%). For CPE isolates majority of the antibiotics were inefficient (Fig 3).

All isolates from blood sample were MDR 11/11 (100%) and 4/11 (36.4%) of them were CPE. 74/95 (77.9%) of urine isolates were MDR and 10/95 (10.5%) of them were CPE. Our finding also showed that 11/13 (84.6%) of Sputum isolates were MDR and 7/13 (53.8%) of them were CPE.

## Discussion

Nowadays, one of the biggest concern globally in health sector is the rise of drug-resistant infections as it poses a serious challenge to public health and modern medicine. For instance, Carbapenem have been utilized recently to manage of several pathogens as last option of treatment; however, many pathogens now produce enzymes that can disable the function of Carbapenem. This growing resistance severely restricts available treatments, leading to increased illness, death rates, and strain on healthcare.

In this study, *E. coli* was the most abundant isolate followed by *K. pneumonia*. The findings was in line with the study conducted in Saudi Arabia [15], Iran [16], Nigeria [17], Debre Birhan [18], Addis Ababa [9]. However, this finding was different from the study done in Sudan [19], where the predominant isolate were *P. mirabilis* followed by *K. pneumoniae*. This discripancy of the fraction of the isolates is likely a function of localized antimicrobial utilization/stewardship, environmental influences, and host factors as well.

In the present study, Multi drug resistance was 78.8%. This finding was consistent with the study done in Bahir Dar (80.0%) [20], (81.1%) [21], Saudi Arabia (70%) [15], Iran (78.31%) [16]. However, the prevalence was lower than a study done in Addis Ababa (94.5%) [22], Gondar, Dessie, Debre Markos collectively (85.8%) [23]. In the contrary, the finding of current study was higher than study done in Addis Ababa (68.6%) [9], (68.3%) [24], (45.2%) [25], Nepal 36.0%, [26]. The observed difference in MDR Prevalence across studies may be attributed to variations in the study population, healthcare settings, antimicrobial utilization practices, infection prevention and control measures and geographical differences.

The predominant MDR isolates in this study were *E.coli* and *K.pneumoniae* and this finding was in line with the study finding in Bahir Dar [20], Saudi Arabia [15], Addis Ababa [22,25]. In contrast, the finding was different from a study done in Nepal [26], which the predominant MDR isolates were *E.coli* and *Citrobacter spp* and from study done in Addis Ababa [9],which, Enterobacter *spp*. and *Citrobacter spp* were the predominant MDR isolates.

In the current study, around 19.5% (30/156) of *Enterobacteriaceae* were carbapenemase producers. This finding was comparable with a study done in Bahir Dar (16.2%) [20], Gondar, Dessie, Debre Markos collectively (15.7%) [23]. However, this result was higher than the findings in study done in Addis Ababa (7.7%) [27], (2%) [22],(5.4%) [25], Bahir Dar(5.2%) [21], (6%) [28], Sidama (9%) [29], Nigeria (7.9%) [17] and Debre Birhan(10.97%) [18].The finding of this study was lower than the study done in Pakistan 24% [30]. The demonstrated difference in Carbapenem resistance is likely a function of methodological variability in enzyme detection and the longitudinal evolution of resistance patterns over time. Plus the variation can be further driven by site-specific selective pressures from antibiotic prescribing habits, the rigor of institutional IPC measures, and laboratory settings.

Moreover, the predominant CPE isolate of present study were *K.pneumoniae* and *E. coli.* This finding was in line with the study done in Addis Ababa [22,25,27] and Debre Birhan [18]. The findings were different from a study done in Bahir Dar [20,21,28]where the predominant isolates were *K. pneumoniae* and E. *cloacae*. Several studies have shown that *K. pneumoniae* and *E. coli* are the predominant and frequents pathogens correlated to this problem as they are also the commonest CRE pathogens in this matter.

In this study, the antimicrobial resistance pattern of CPE isolates indicates that all CPE isolates were 100% resistant to ampicillin, amoxicillin-clavulanic acid, ceftriaxone, cefepime, co-trimoxazole, 96.6% resistance to ciprofloxacin and 80% to Amikacin. This was different from a study done in Addis Ababa with 100%resistance to ampicillin, amoxicillin with clavulanic acid, 87.5% resistance to, sulfamethoxazole-trimethoprim and 75.0% resistance to ceftriaxone and cefepime [9]. This extensive resistance profile of CRE of the isolates could be driven by other factors like ESBLs and carbapenemases frequently localized on the same conjugative plasmids as genes conferring resistance to aminoglycosides and fluoroquinolones.

Furthermore, there are limited treatment options for CPE isolates with Amikacin being the least resistant drug with resistance of 80%. This finding was much higher compared to the study done in Addis Ababa where no resistance to amikacin detected for CPE isolates [9]. This might be due to the acquisition of resistance gene over time.

The strength of the study was utilization of MALDI-TOF for identification purpose as this advanced diagnostic method enabled accurate and rapid identification of *Enterobacteriaceae* isolates. In addition, the magnitude of CPE and MDR was studied in a relatively larger number of clinical specimens. On the other hands, the limitation was differentiation of Salmonella isolates to the species level were difficult due to the constraints of the MALDI-TOF MS database.

## Conclusion

A high proportion of isolates showed multidrug resistance, highlighting the growing challenge of antimicrobial resistance in clinical settings. Furthermore, detection of increased prevalence carbapenemase-producing *Enterobacteriaceae* is alarming, which further limits therapeutic options and patient outcome. *Klebsiella pneumonia* was the most common carbapenemase producer. Highest resistance was noted against ampicillin, amoxicillin-clavulanic acid, co-trimoxazole, and ciprofloxacin. As a result, these findings underscore the urgent need for robust antimicrobial stewardship, early detection strategies, and reinforced infection control program. Furthermore, practitioners should recognize the burden of CPE originating from private healthcare facilities, and interventions aimed at public institutions should also take private facilities into account to effectively address the issue.

## Abbreviations

AR: Antibiotic resistance
ATCC: American Type Culture Collection
CLSI: Clinical Laboratory standards Institute
CPE: Carbapenemase producing *Enterobacteriaceae*
CRE: Carbapenem resistant *Enterobacteriaceae*
ESBL: Extended spectrum β-lactamases
IPC: Infection prevention and control
MALDI-TOF: Matrix-Assisted Laser Desorption & Ionization- Time of Flight
MDR: Multidrug-resistant
mCIM: , Modified carbapenem inactivation method
SOP: Standard operating procedure
Spp: Species
WAML: Wudassie Advanced Medical Laboratory

## References

[1] Medina E, Pieper DH. Tackling threats and future problems of multidrug-resistant bacteria. Curr Top Microbiol Immunol. 2016;398:3–33. doi: 10.1007/82_2016_492 27406189

[2] Prestinaci F, Pezzotti P, Pantosti A. Antimicrobial resistance: a global multifaceted phenomenon. Pathog Glob Health. 2015;109(7):309–18. doi: 10.1179/2047773215Y.0000000030 26343252 PMC4768623

[3] Kohlenberg A, Weitzel-Kage D, van der Linden P, Sohr D, Vögeler S, Kola A, et al. Outbreak of carbapenem-resistant Pseudomonas aeruginosa infection in a surgical intensive care unit. J Hosp Infect. 2010;74(4):350–7. doi: 10.1016/j.jhin.2009.10.024 20170982

[4] Antimicrobial Resistance Collaborators. Global burden of bacterial antimicrobial resistance in 2019: a systematic analysis. Lancet. 2022;399(10325):629–55. doi: 10.1016/S0140-6736(21)02724-0 35065702 PMC8841637

[5] Cassini A, Högberg LD, Plachouras D, Quattrocchi A, Hoxha A, Simonsen GS, et al. Attributable deaths and disability-adjusted life-years caused by infections with antibiotic-resistant bacteria in the EU and the European Economic Area in 2015: a population-level modelling analysis. Lancet Infect Dis. 2019;19(1):56–66. doi: 10.1016/S1473-3099(18)30605-4 30409683 PMC6300481

[6] Morrill HJ, Pogue JM, Kaye KS, LaPlante KL. Treatment Options for Carbapenem-Resistant Enterobacteriaceae Infections. Open Forum Infect Dis. 2015;2(2):ofv050. doi: 10.1093/ofid/ofv050 26125030 PMC4462593

[7] Alemayehu T. Prevalence of multidrug-resistant bacteria in Ethiopia: a systematic review and meta-analysis. J Glob Antimicrob Resist. 2021;26:133–9. doi: 10.1016/j.jgar.2021.05.017 34129993

[8] Alemayehu E, Fiseha T, Gedefie A, Alemayehu Tesfaye N, Ebrahim H, Ebrahim E, et al. Prevalence of carbapenemase-producing Enterobacteriaceae from human clinical samples in Ethiopia: a systematic review and meta-analysis. BMC Infect Dis. 2023;23(1):277. doi: 10.1186/s12879-023-08237-5 37138285 PMC10155349

[9] Tekele SG, Teklu DS, Legese MH, Weldehana DG, Belete MA, Tullu KD, et al. Multidrug-Resistant and Carbapenemase-Producing Enterobacteriaceae in Addis Ababa, Ethiopia. Biomed Res Int. 2021;2021:9999638. doi: 10.1155/2021/9999638 34195291 PMC8214486

[10] Wilson ML. General principles of specimen collection and transport. Clin Infect Dis. 1996;22(5):766–77. doi: 10.1093/clinids/22.5.766 8722929

[11] Singhal N, Kumar M, Kanaujia PK, Virdi JS. MALDI-TOF mass spectrometry: an emerging technology for microbial identification and diagnosis. Front Microbiol. 2015;6:791. doi: 10.3389/fmicb.2015.00791 26300860 PMC4525378

[12] Performance Standards for Antimicrobial Susceptibility Testing. https://clsi.org/shop/standards/m100/. Accessed 2025 December 6.

[13] Magiorakos A-P, Srinivasan A, Carey RB, Carmeli Y, Falagas ME, Giske CG, et al. Multidrug-resistant, extensively drug-resistant and pandrug-resistant bacteria: an international expert proposal for interim standard definitions for acquired resistance. Clin Microbiol Infect. 2012;18(3):268–81. doi: 10.1111/j.1469-0691.2011.03570.x 21793988

[14] Carbapenemase Producing Carbapenem-Resistant Enterobacteriaceae (CP-CRE) 2018 Case Definition. 2018. Available from: https://ndc.services.cdc.gov/case-definitions/carbapenemase-producing-carbapenem-resistant-enterobacteriaceae-2018/

[15] Bandy A, Tantry B. ESBL Activity, MDR, and Carbapenem Resistance among Predominant Enterobacterales Isolated in 2019. Antibiotics (Basel). 2021;10(6):744. doi: 10.3390/antibiotics10060744 34205425 PMC8234840

[16] Mirzaei B, Babaei R, Bazgir ZN, Goli HR, Keshavarzi S, Amiri E. Prevalence of Enterobacteriaceae spp. and its multidrug-resistant rates in clinical isolates: A two-center cross-sectional study. Molecular Biology Reports. 2021;48(1):665–75. doi: 10.1007/s11033-020-06114-x 33389531

[17] Aminu A, Daneji IM, Yusuf MA, Jalo RI, Tsiga-Ahmed FI, Yahaya M, et al. Carbapenem-resistant Enterobacteriaceae infections among patients admitted to intensive care units in Kano, Nigeria. Sahel Medical J. 2021;24(1):1–9. doi: 10.4103/smj.smj_14_20

[18] Shibabaw A, Sahle Z, Metaferia Y, Atlaw A, Adenew B, Gedefie A, et al. Epidemiology and prevention of hospital-acquired carbapenem-resistant Enterobacterales infection in hospitalized patients, Northeast Ethiopia. IJID Reg. 2023;7:77–83. doi: 10.1016/j.ijregi.2023.02.008 37009574 PMC10050477

[19] Almugadam BS, Saeed Elbala A, ELdeen Elkheir AS, Abdul Mazid M, Osman SA. Carbapenem resistance enterobacteriaceae among wound isolates, Kosti City, Sudan. Clin Microbiol. 2018;07(01). doi: 10.4172/2327-5073.1000305

[20] Moges F, Eshetie S, Abebe W, Mekonnen F, Dagnew M, Endale A, et al. High prevalence of extended-spectrum beta-lactamase-producing Gram-negative pathogens from patients attending Felege Hiwot Comprehensive Specialized Hospital, Bahir Dar, Amhara region. PLoS One. 2019;14(4):e0215177. doi: 10.1371/journal.pone.0215177 30986262 PMC6464180

[21] Alebel M, Mekonnen F, Mulu W. Extended-Spectrum β-Lactamase and Carbapenemase Producing Gram-Negative Bacilli Infections Among Patients in Intensive Care Units of Felegehiwot Referral Hospital: A Prospective Cross-Sectional Study. Infect Drug Resist. 2021;14:391–405. doi: 10.2147/IDR.S292246 33564247 PMC7867495

[22] Beyene D, Bitew A, Fantew S, Mihret A, Evans M. Multidrug-resistant profile and prevalence of extended spectrum β-lactamase and carbapenemase production in fermentative Gram-negative bacilli recovered from patients and specimens referred to National Reference Laboratory, Addis Ababa, Ethiopia. PLoS One. 2019;14(9):e0222911. doi: 10.1371/journal.pone.0222911 31553773 PMC6760794

[23] Moges F, Gizachew M, Dagnew M, Amare A, Sharew B, Eshetie S, et al. Multidrug resistance and extended-spectrum beta-lactamase producing Gram-negative bacteria from three Referral Hospitals of Amhara region, Ethiopia. Ann Clin Microbiol Antimicrob. 2021;20(1):16. doi: 10.1186/s12941-021-00422-1 33706775 PMC7953565

[24] Teklu DS, Negeri AA, Legese MH, Bedada TL, Woldemariam HK, Tullu KD. Extended-spectrum beta-lactamase production and multi-drug resistance among Enterobacteriaceae isolated in Addis Ababa, Ethiopia. Antimicrob Resist Infect Control. 2019;8:39. doi: 10.1186/s13756-019-0488-4 30815254 PMC6377715

[25] Abdeta A, Bitew A, Fentaw S, Tsige E, Assefa D, Lejisa T, et al. Phenotypic characterization of carbapenem non-susceptible gram-negative bacilli isolated from clinical specimens. PLoS One. 2021;16(12):e0256556. doi: 10.1371/journal.pone.0256556 34855767 PMC8638961

[26] Basnet A, Shrestha MR, Tamang B, Pokhrel N, Maharjan R, Rai JR, et al. Assessment of Antibiotic Resistance among Clinical Isolates of Enterobacteriaceae in Nepal. Am J Trop Med Hyg. 2024;110(2):283–90. doi: 10.4269/ajtmh.23-0199 38167427 PMC10859788

[27] Seman A, Mihret A, Sebre S, Awoke T, Yeshitela B, Yitayew B, et al. Prevalence and Molecular Characterization of Extended Spectrum β-Lactamase and Carbapenemase-Producing Enterobacteriaceae Isolates from Bloodstream Infection Suspected Patients in Addis Ababa, Ethiopia. Infect Drug Resist. 2022;15:1367–82. doi: 10.2147/IDR.S349566 35378892 PMC8976516

[28] Tadesse S, Mulu W, Genet C, Kibret M, Belete MA. Emergence of High Prevalence of Extended-Spectrum Beta-Lactamase and Carbapenemase-Producing Enterobacteriaceae Species among Patients in Northwestern Ethiopia Region. Biomed Res Int. 2022;2022:5727638. doi: 10.1155/2022/5727638 35155675 PMC8837423

[29] Alemayehu T, Asnake S, Tadesse B, Azerefegn E, Mitiku E, Agegnehu A, et al. Phenotypic Detection of Carbapenem-Resistant Gram-Negative Bacilli from a Clinical Specimen in Sidama, Ethiopia: A Cross-Sectional Study. Infect Drug Resist. 2021;14:369–80. doi: 10.2147/IDR.S289763 33564245 PMC7866937

[30] Mustafai MM, Hafeez M, Munawar S, Basha S, Rabaan AA, Halwani MA, et al. Prevalence of Carbapenemase and Extended-Spectrum β-Lactamase Producing Enterobacteriaceae: A Cross-Sectional Study. Antibiotics (Basel). 2023;12(1):148. doi: 10.3390/antibiotics12010148 36671350 PMC9854900

